# Relationship of muscle morphology to hip displacement in cerebral palsy: a pilot study investigating changes intrinsic to the sarcomere

**DOI:** 10.1186/s13018-019-1239-1

**Published:** 2019-06-21

**Authors:** Kelly A. Larkin-Kaiser, Jason J. Howard, Timothy Leonard, Venus Joumaa, Luke Gauthier, Karl Logan, Benjamin Orlik, Ron El-Hawary, Walter Herzog

**Affiliations:** 1 Faculty of Kinesiology, University of Calgary, 376 Collegiate Blvd NW, Calgary, AB T2N 4V8 Canada; 2 IWK Health Centre, 5980 University Ave, Halifax, NS B3K 6R8 Canada; 3 Weill Cornell Medicine, Sidra Medicine, Al Gharrafa St, Ar Rayyan, P.O. Box 26999, Doha, Qatar

**Keywords:** Hip displacement, Cerebral palsy, Sarcomere, Titin, Adductor muscles

## Abstract

**Background:**

Cerebral palsy (CP) is the most common cause of childhood disability, typified by a static encephalopathy with peripheral musculoskeletal manifestations—most commonly related to spasticity—that are progressive with age. Hip displacement is one of the most common manifestations, observed to lead to painful degenerative arthritis over time. Despite the key role that spasticity-related adductor muscle contractures are thought to play in the development of hip displacement in CP, basic science research in this area to date has been limited. This study was initiated to correlate hip adductor muscle changes intrinsic to the sarcomere—specifically, titin isoforms and sarcomere length—to the severity of hip displacement in children with spastic cerebral palsy.

**Methods:**

Single gracilis muscle biopsies were obtained from children with CP (Gross Motor Function Classification System (GMFCS) III-V; *n* = 10) who underwent adductor muscle release surgery for the treatment of hip displacement. Gel electrophoresis was used to estimate titin molecular weight. Sarcomere lengths were measured from muscle fascicles using laser diffraction. The severity of hip displacement was determined by measuring by Reimers migration percentage (MP) from anteroposterior pelvic x-rays. Correlation analyses between titin, sarcomere lengths, and MP were performed.

**Results:**

The mean molecular weight of titin was 3588 kDa. The mean sarcomere length was 3.51 μm. Increased MP was found to be associated with heavier isoforms of titin (*R*^2^ = 0.65, *p* < 0.05) and with increased sarcomere lengths (*R*^2^ = 0.65, *p* < 0.05). Heavier isoforms of titin were also associated with increased sarcomere lengths (*R*^2^ = 0.80, *p* < 0.05).

**Conclusions:**

Our results suggest that both larger titin isoforms and sarcomere lengths are positively correlated with increased severity of hip displacement and may represent adaptations in response to concomitant increases in spasticity and muscle shortening.

**Trial registration:**

As this study does not report the results of a health care intervention on human participants, it has not been registered.

## Introduction

**Fig. 1.**
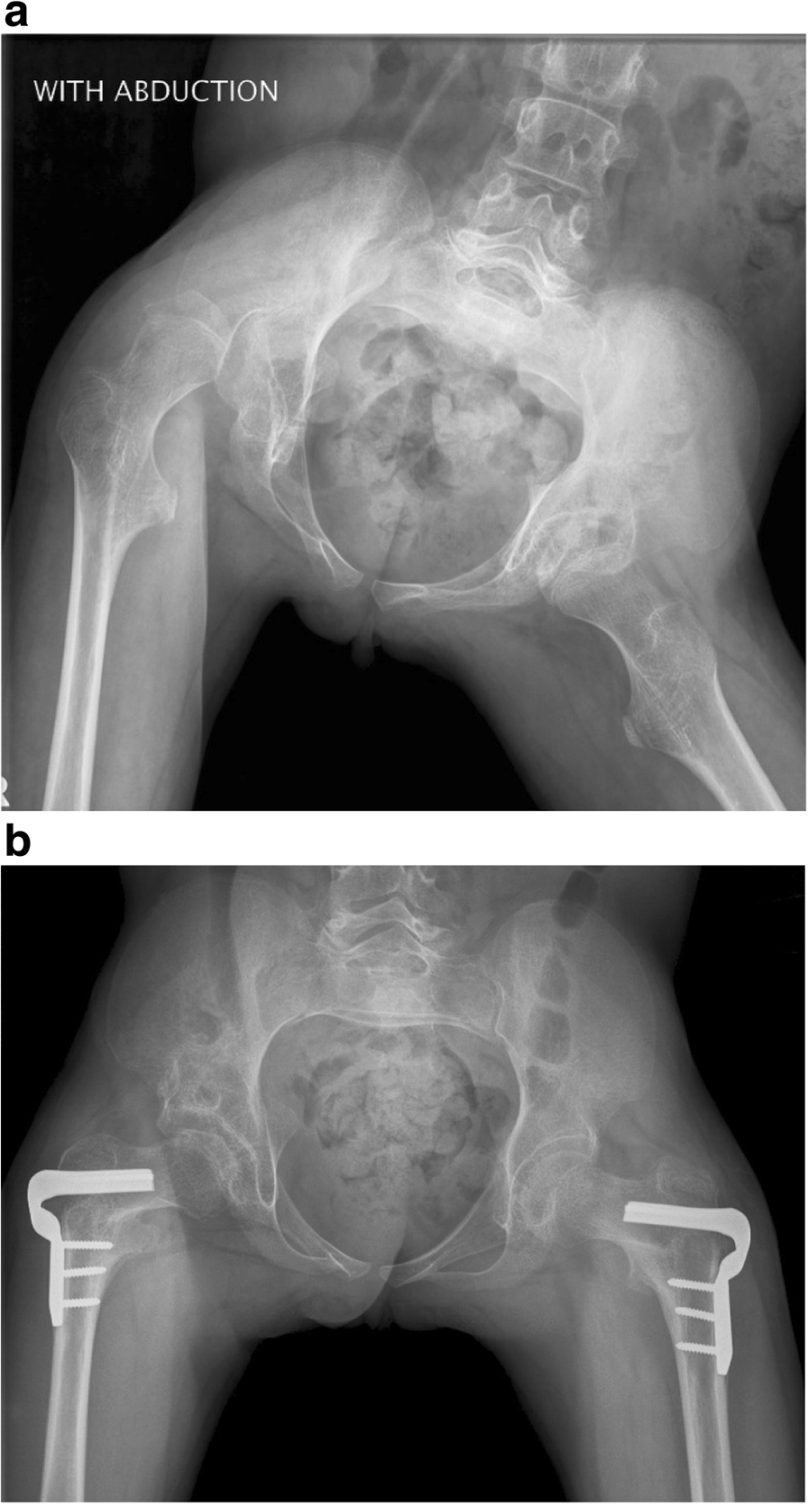
**a** 9-year-old girl with spastic quadriplegic CP (GMFCS level IV) with a right hip dislocation and windswept hip deformity. **b** Post-operative bilateral hip adductor and flexor lengthenings (adductor longus, gracilis, iliopsoas), bilateral proximal femoral varus derotational osteotomies, right San Diego acetabuloplasty. Both hips are now concentrically reduced with improvements of pain and care-giving realized (CP, cerebral palsy; GMFCS, gross motor functional classification system)

Cerebral palsy (CP) is the most common cause of childhood disability, occurring in 2 to 3 out of 1000 live births [1]. CP is a spectrum disorder that describes a neurological compromise secondary to an insult in the developing brain. Although CP results from a static encephalopathy, the peripheral musculoskeletal manifestations are progressive with age. Spastic CP is most common and presents with a velocity dependent (dynamic) increase in muscle stiffness that is thought to precede the development of (static) muscle contracture. These static contractures, in turn, are associated with limitations in joint range of motion (ROM), secondary bony deformities, and, in the case of the hip, progressive displacement (joint subluxation and/or dislocation). The incidence of hip displacement in CP has been found to be linearly related to increasing disease severity as stratified by the Gross Motor Function Classification System (GMFCS). In a population-based study, the incidence of hip displacement ranged from 0% for patients in GMFCS I to 89% in GMFCS V, with an overall incidence of 35% for all GMFCS levels [2]. The natural history of hip displacement in CP has been observed to lead to painful degenerative arthritis. As such, surgical interventions are often required in the form of muscle lengthenings (typically hip adductors, hip flexors, and proximal hamstrings), bony reconstruction of the femur, and/or acetabulum [3] (Fig. 1).

Despite the key role that spasticity-related muscle contractures are thought to play in the development of hip displacement in CP, basic science research in this area to date has been limited. More specifically, the development of intrinsic cellular pathoanatomical changes to the sarcomere have not been explored thoroughly enough to identify an altered phenotype associated with CP. Muscle myofibrils are sub-cellular organelles comprised of long, serially arranged sarcomeres, each containing actin (thin) and myosin (thick) myofilaments which slide over one another during muscle contraction according to the sliding filament theory[4]. Previous research has found the sarcomere, the basic contractile unit in skeletal muscle, is elongated in spastic muscle tissue. In fact, intraoperative sarcomere length analysis has revealed that CP muscle tissue consists of extremely long resting sarcomere lengths, even though the muscles themselves were shortened overall [5–9]. Indeed, it has been suggested that muscle weakness in CP—associated with long sarcomeres with decreased force generating capacity—rather than spasticity-related muscle shortening, may be the real driver of musculoskeletal pathologies such as hip displacement [10]. Sarcomere length changes relating to the severity of hip displacement, however, have not yet been explored or reported.

Myosin filaments within the sarcomere are stabilized and tethered to the Z-line at the end of each sarcomere by the molecular spring titin. Titin is a large molecule said to be responsible for most of the passive stiffness of the myofibril [11, 12] and has been shown to increase myofibrillar stiffness due to calcium binding upon muscle activation [12]. Given these proposed functions, it is reasonable to explore titin’s role in, or adaptation to, the development of muscle contracture in CP, and ultimately, clinical dysfunction.

Given that the incidence of hip displacement in spastic CP is very high (approximately 35% [2]) and has been found to be directly related to disease severity, this patient group is appropriate for studying the relationship between muscle morphology and clinical dysfunction. Although performed for decades, surgical lengthening of muscles in the hip adductor-flexor group (most commonly, gracilis, adductor longus, and iliopsoas) for hip displacement in CP has not been shown to reliably achieve surgical success, particularly in children of increased disease severity [13]. As such, the current study was initiated to identify and correlate structural muscle changes intrinsic to the sarcomere (specifically, titin isoform and sarcomere length) within the hip adductors (gracilis) to disease severity in children undergoing soft tissue releases for progressive hip displacement in spastic CP. Understanding the pathophysiological mechanisms underlying the development of spastic muscle contracture as it relates to disease severity in CP will help inform the most appropriate choice of treatment and/or the future development of novel interventions for the management of spasticity-related muscle contractures associated with cerebral palsy [14].

## Materials and methods

### Study design and participant selection

Consecutive patients with CP and a primarily spastic motor type (GMFCS III to V) were prospectively enrolled. All patients subsequently underwent elective tendon releases for the treatment of dynamic and/or static muscle contractures associated with progressive hip displacement (+\− bony procedures as indicated). Children were recruited from the institution’s multi-disciplinary Cerebral Palsy Clinic at a tertiary level academic children’s hospital. Children who had previous surgery of the hip adductor muscle group or had undergone botulinum toxin injection into the target muscle(s) within 6 months pre-operatively were excluded. Children with a primarily non-spastic motor type were also excluded.

### Pre-operative measures

All clinical and radiographic measures were recorded pre-operatively and included GMFCS level, hip range of motion (goniometry), spasticity measures (Modified Tardieu Scale, Ashworth Scale), and radiographic measures (Reimers hip migration percentage (MP), Melbourne CP Hip Classification System (MCPHCS) [15]). Each child’s gross motor function was classified according to the GMFCS by the lead pediatric neurologist associated with the institution’s Cerebral Palsy Clinic [16]. This neurologist also confirmed the participant’s motor type to be primarily spastic.

**Fig. 2:**
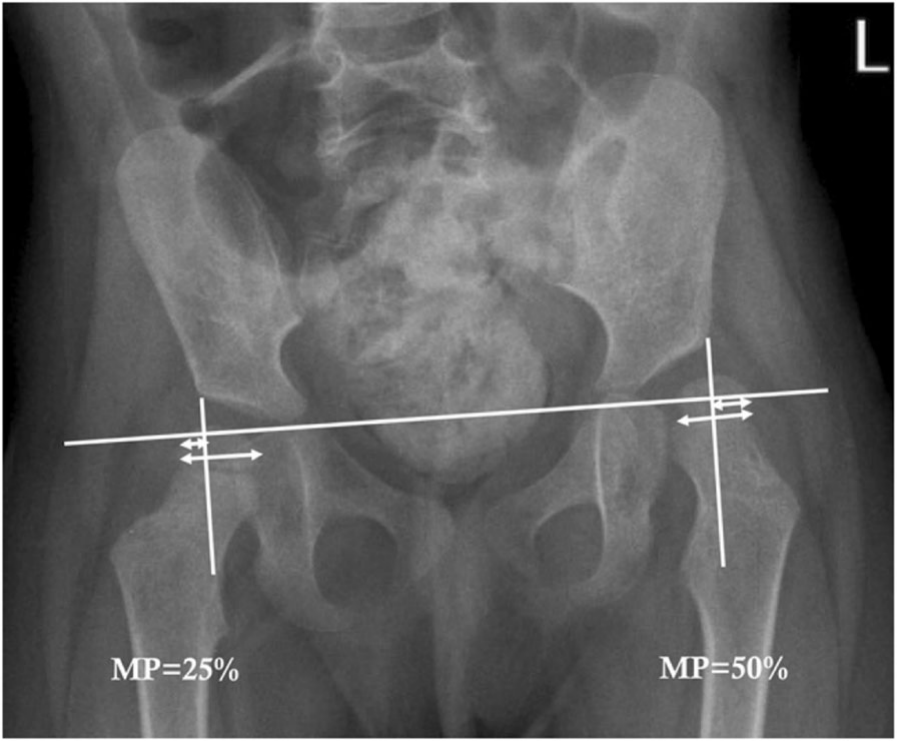
Quantification of hip displacement (subluxation or dislocation) by migration percentage (MP) for children with cerebral palsy. MP is validated measure calculated through a ratio of the extent of the femoral head lateral to the edge of the acetabulum (delineated by the vertical Perkin’s line shown) divided by the diameter of the femoral head. The measurements should be parallel to the horizontal Hilgenreiner’s line shown (a line connecting the superior aspects of the triradiate cartilages)

Participants were indicated for surgical intervention according to the discretion of the treating surgeon primarily using the results of the MP, a validated radiographic measure of the percentage of displacement of the femoral head from the acetabulum [17] (Fig. 2). Typically, an MP of greater than 30% that is progressive or an absolute MP greater than 40% was taken as an indication for surgery. MP was recorded from pre-operative anteroposterior pelvic X-rays. According to Robin and colleagues, a MP of less than 10% is considered to be normal, a MP of greater than, or equal to, 10% is considered to be subluxated, and a MP of 100% is classified as dislocated [15].

On the morning of the surgery, each participant underwent a physical examination by one of the study’s co-authors (LG) to determine the presence of dynamic and/or static hip abduction contractures. This examiner was specially trained to perform these clinical measurements by a senior pediatric orthopedic surgeon with sub-specialty clinical fellowship training and extensive expertise in the assessment and treatment of children with cerebral palsy (JJH). With the participant in the supine position, maximum hip abduction was measured for two positions: with the hip and knee at 90° flexion and with the hip and knee at 0° flexion. These positions represented the contributions of the adductor longus and the proximal hamstrings (including gracilis), respectively. These measures were obtained by goniometric measurement. To determine the presence of dynamic and/or static contractures, measurement was performed according to the Modified Tardieu Scale, whereby the angle of maximum hip abduction was taken at the point of “spastic catch” after the joint was moved in a quick stretch (R1) and after the joint was moved slowly to its end point (R2), respectively. The muscle tone of the CP participants was described using the Ashworth Scale [18].

### Muscle biopsy procedure

Under general anesthesia, biopsies of the adductor longus and gracilis were performed during the course of a soft tissue release procedure for the treatment of hip displacement. The surgical approach was via a medial (i.e., groin) approach, centered on the adductor longus, using a transverse incision of approximately 3 to 4 cm in length. The deep fascia overlying the adductor longus was incised in line with the incision and the muscles under investigation exposed with biopsies being taken of the adductor longus and gracilis. Small samples of operated muscle were excised by the treating orthopedic surgeon (3 mm length, 3 mm diameter), using a specially designed single-use polypropylene biopsy clamp. Experience from our laboratory indicates that sarcomere lengths obtained using these biopsy clamps versus fixing the muscle sample directly in situ (gold standard) did not result in significant differences between the two methods. The biopsy clamp was utilized in order to preserve the in situ muscle and sarcomere lengths at the muscle’s end range. With the hip in maximum (but not forced) abduction, the adductor longus and gracilis were biopsied with the hip and knee at 90° flexion and 0° flexion, respectively. The degree of intra-operative hip abduction at the time of sampling was also recorded. Although both the adductor longus and gracilis were biopsied, the latter muscle was analyzed for the purposes of this study. The adductor longus specimens underwent a separate analysis which are the subject of another study.

### Sarcomere length measurement

Following biopsy, the muscle tissue was submerged in a 10% formalin solution and stored for 30 days to allow for adequate fixation of the sarcomeres. After fixation, the tissue was removed from the formalin solution and immersed in a solution of 30% nitric acid and 70% distilled water for approximately 12 h. Following digestion of the extracellular matrix, the tissue was washed in a saline solution for 2 h and then placed in glycerol. Ten individual muscle fascicles were isolated from each muscle biopsy, mounted onto a glass slide and covered with a coverslip. Mean sarcomere lengths were then obtained from 5 measurements along the length of each fascicle using laser diffraction (beam diameter = 0.8 mm) [19, 20].

### Titin gel electrophoresis

**Fig. 3:**
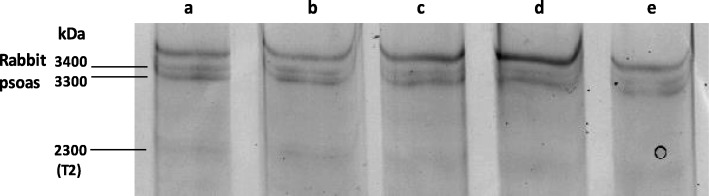
Titin molecular weight determination. Two percent of agarose strengthened gel loaded with gracilis muscle from children with spastic cerebral palsy and normal rabbit psoas muscles used as internal molecular weight markers. Rabbit psoas muscle was used as an internal molecular weight marker. Wells **a**–**e** are extracts from CP tissue (top band) and rabbit psoas muscle. T2 is a proteolytic product of intact titin

All muscle samples were snap-frozen in liquid nitrogen immediately following biopsy. Frozen samples were solubilized in a buffer containing 4.3 mM Tris (pH 6.8), 4.3 mM EDTA, 1% SDS, 1% 2-β mercaptoethanol, 10% glycerol, 0.1% bromophenol blue, and 4 μg/ml leupeptin. Samples were incubated for 5 min on ice, boiled for 3 min, and then centrifuged for 15 min at 14,000×*g*. Titin molecular weight was determined using 2% agarose strengthened SDS polyacrylamide gels with a Laemmli buffer system. The gels were run in a Biorad Mini-Protean Tetra Cell unit (Bio-Rad Laboratories Inc, CA, USA) at a constant voltage of 22 V overnight at room temperature. The gels were then stained with Coomassie Blue and scanned using a Biorad GS-800 densitometer (Bio-Rad Laboratories Inc, CA, USA). To estimate the molecular weight of titin isoforms in the CP muscles, each well of the gel was loaded with a cerebral palsy muscle sample of unknown titin isoform and rabbit psoas muscle sample which expresses two N2A titin isoforms of molecular weights 3416 and 3295 kDa (Fig. 3). In order to test the accuracy of our molecular weight measurements, we determined titin molecular weight in rabbit soleus muscle and rat heart and compared it to previously reported results (3600 and 3000 kDa for rabbit soleus and rat heart respectively) [21, 22]. Measurements of titin molecular weight were done using ImageJ software [23]. Mean values (± SEM) of titin molecular weights were calculated from three to six observations per muscle sample.

### Outcome measures

The primary outcome measure was titin molecular weight correlated to MP. Secondary outcome measures included sarcomere length measurement, pre-operative hip abduction goniometry (R1 and R2), intra-operative hip abduction goniometry, MP, and MCPHCS level.

### Statistical analysis

Tissue samples were utilized for titin isoform analysis and the corresponding titin molecular weights were correlated with their respective MPs using multiple regression analysis.

To determine whether sarcomere lengths observed in CP muscle tissue were associated with structural myofibrillar changes and proxies of clinical dysfunction, sarcomere lengths were correlated to titin isoform molecular weights and to MP using multiple regression analysis.

In addition, post hoc power analyses were performed to determine statistical power.

This analysis was performed in consultation with a statistician, using SPSS v.18 (IBM Corp., Armonk, NY, USA).

## Results

Bilateral biopsy samples from gracilis were obtained from 10 children with spastic CP who met the study inclusion criteria. Of the 10 biopsies, measurable results were obtained from 7 samples for titin analysis and 9 for sarcomere length analysis. The mean age of the participants was 8.8 ± 4.2 years (range, 2 to 12 years). With respect to functional level, 10%, 70%, and 20% of participants were GMFCS III, IV, and V, respectively.

### Clinical measures

The mean pre-operative hip abduction (with knee and hip at 0° flexion) was 11° (range, − 10 to 30°) and 25° (range, − 5 to 60°) for R1 and R2, respectively. The mean intraoperative hip abduction (with knee and hip at 0° flexion) was 32° (range, 15 to 50°). The mean pre-operative hip abduction (with knee and hip at 90° flexion) was 20° (range, 5 to 45°) and 29° (range, 15 to 60°) for R1 and R2, respectively. The mean intraoperative hip abduction (with knee and hip at 90° flexion) was 36° (range, 15 to 65°).

With respect to Ashworth Scale ratings for the CP Group, 30% were rated as 3, 60% as 2, and 10% as 1.

With respect to radiographic measures, the mean pre-operative MP was 44% (range, 15 to 86%). According to the MCPHCS, 45% of hips were rated as level 4 (subluxated), 45% as level 3 (dysplastic), and 10% as level 2 (near normal hip).

### Titin molecular weight

**Fig. 4:**
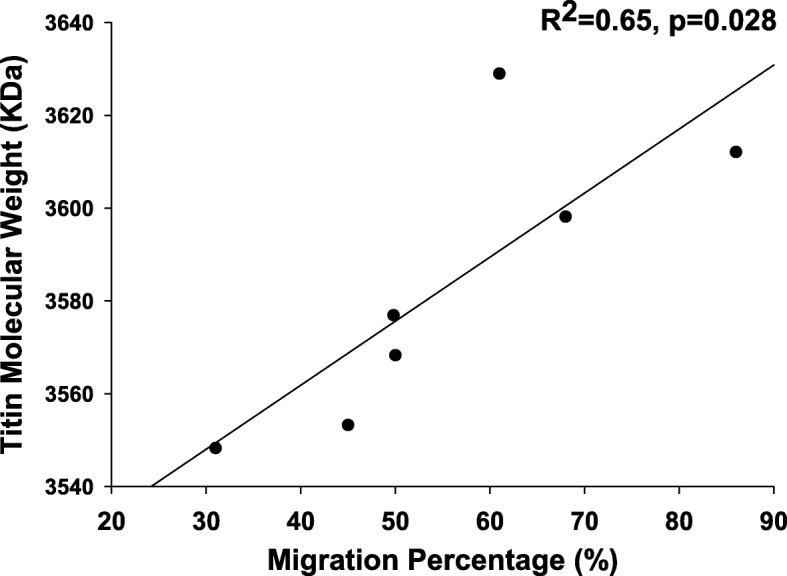
Correlation between hip migration percentage measured for CP subjects and the titin isoform molecular weight expressed in the gracilis muscle. We found a positive correlation (*p* = 0.028), meaning subjects with more severe hip displacements also had larger isoforms of titin expressed

The average molecular weight of titin was found to be 3583 ± 30 kDa (range, 3548 to 3629 kDa). Titin molecular weight was found to be significantly correlated to the severity of hip displacement (by MP, *R*^2^ = 0.65, *p* = 0.028, Fig. 4). Post hoc power analysis revealed an observed statistical power > 0.9.

### Sarcomere length measurement

**Fig. 5.**
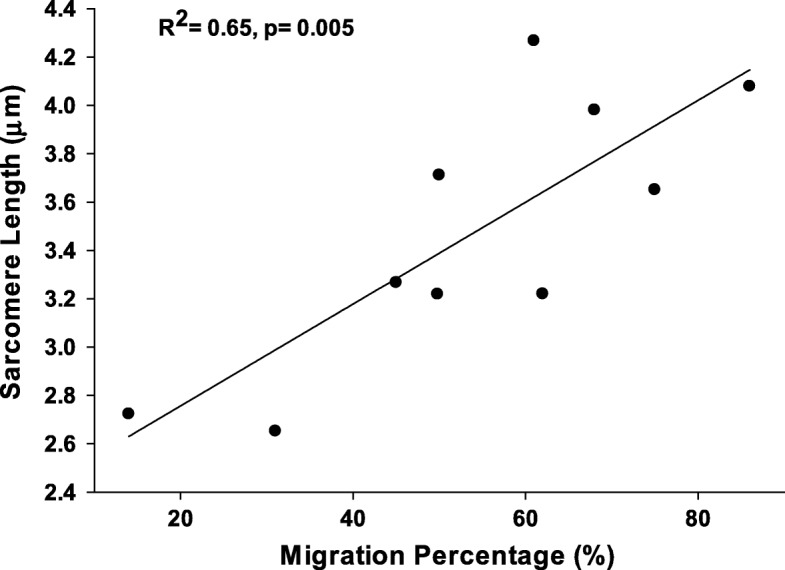
Correlation between in vivo sarcomere lengths for gracilis and the degree of hip displacement as quantified by MP. We found a positive correlation (*p* = 0.005), meaning that CP subjects with longer in vivo sarcomere lengths also had more severe hip displacement

**Fig. 6:**
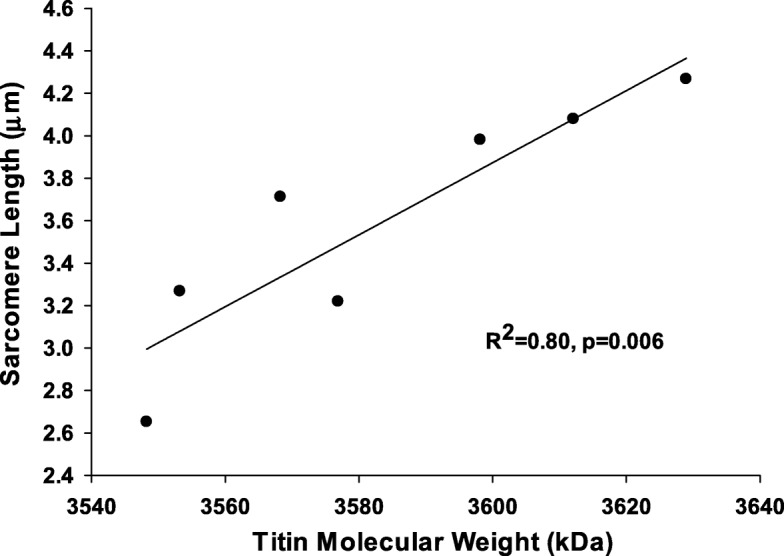
Correlation between in vivo sarcomere lengths for CP subjects and the titin isoform weight expressed in gracilis. We found a positive correlation (*p* = 0.006), meaning that subjects with longer in vivo sarcomere lengths also had larger isoforms of titin expressed in their gracilis muscle

The mean in vivo gracilis sarcomere length was found to be 3.51 ± 0.05 μm. There was a significant correlation between increased in vivo sarcomere length and the severity of hip displacement (by MP, *R*^2^ = 0.65, *p* = 0.005, Fig. 5). In addition, in vivo sarcomere lengths were significantly correlated to larger titin isoforms (*R*^2^ = 0.80, *p* = 0.006; Fig. 6). Post hoc power analysis revealed an observed statistical power > 0.9.

## Discussion

In this pilot study, we measured titin molecular weights and sarcomere lengths from the gracilis muscle of children with spastic CP to help identify pathoanatomical changes in the sarcomere relating to clinical dysfunction, more specifically, hip displacement. While previous studies have reported increased sarcomere lengths in spastic muscles, the corresponding data on mean titin isoform weights have found no significant difference between patients with CP and typically developing controls [8, 24]. In the current study, however, we found a significant positive correlation between titin molecular weight and hip displacement according to MP. Given titin’s role in regulating myofibrillar stiffness within the sarcomere, this relationship may represent an adaptive feature in response to increases in dynamic and static muscle contractures associated with increases in hip displacement. The specifics of this adaptive response were not investigated in this study and have yet been elucidated.

For increasing severity of hip displacement, we also found a significant positive correlation between sarcomere length and MP. This suggests that, like titin, increases in sarcomere length may contribute to the development of clinical dysfunction associated with hip subluxation/dislocation, or may present as an adaptation in response to the spasticity and overall muscle shortening typically found in CP. This relationship between clinical dysfunction and increases in sarcomere length has been previously suggested. In an in vivo study of CP patients across all GMFCS levels undergoing hamstring lengthening surgery, for both the gracilis and semitendinosus, Smith et al. demonstrated a significant negative correlation between popliteal angle and in vivo sarcomere length, indicating that longer sarcomere lengths were associated with increased contracture severity [24]. In this same study, increased sarcomere lengths demonstrated a significant positive correlation to the GMFCS, a more global measure of overall motor function for children with CP. The results of our pilot study are consistent with this previous research, specifically with respect to the gracilis muscle.

Performing hip adductor muscle releases via a medial (i.e., groin) approach—as is typical for the treatment of hip displacement in CP—can be difficult depending on the extent of static muscle contracture present. In this study, the end range of passive hip abduction was typically short but variable, which made it difficult to biopsy the muscles at a predefined joint angle for all specimens. In the gracilis (hip and knee flexion at 0°, mean intraoperative abduction angle, 32°), for example, we found that the end range of intraoperative abduction was a minimum of 15° and a maximum of 50° for all participants. As such, choosing a predefined joint angle at the time of biopsy (in an attempt to normalize sarcomere lengths between subjects, 15° for the purposes of this study) would mean that those with less static contracture tended to be substantially slack (grossly) compared to those with increased static contracture (i.e., less hip abduction) at the time of muscle biopsy. Previous studies have found that there was a substantial decrease in the variability of slack sarcomere lengths for muscles near their end range compared to muscles that are in their mid-range of excursion [25–27]. In addition, sarcomere length variability has been shown to decrease significantly when the muscle is under passive tension (closer to its end range) at the time of measurement [25]. Given that the gracilis in the CP group had substantial variability in muscle slackness at a given joint angle, due to differences in the extent of static contracture present for each, there were some concerns that the inherent variability in sarcomere lengths would make the interpretation of our in vivo sarcomere lengths problematic.

Given these concerns, it was decided to take a biopsy with the muscles at maximum (but not forced) hip abduction to facilitate surgical access and to allow the muscles to be at more uniform levels of gross muscle tension. This was done to help allow for the measurement of sarcomere length at the end range for these muscles (with varying amounts of static contracture) and better standardize the passive tension imparted. The aim of this approach was to decrease the variability of sarcomere lengths due to muscle slackness at a pre-defined common joint angle at the time of biopsy for all participants. In support of our approach, the mean in vivo gracilis sarcomere length in our study was found to be 3.51 ± 0.05 μm, comparable to the results of Smith and colleagues, who found a mean sarcomere length of 3.54 ± 0.14 μm for the gracilis in CP children undergoing hamstring lengthening surgery, considerably longer sarcomere lengths as compared to typically developing children [24]. In further support, the relationship between the joint angle at which the biopsy of gracilis was taken and the sarcomere length measured was examined for a relationship, but no significant correlation was found.

Muscles are believed to operate near optimal sarcomere lengths of approximately 2.5 to 2.7 μm in human skeletal muscle [28, 29]. The mean sarcomere length found in CP children (approximately 3.5 μm), however, is halfway down on the descending limb of the force-length curve [30]. As such, these elongated sarcomeres may be associated with a loss of active force generating ability (i.e., weakness) often seen in children with CP [31–33].

With increases in titin molecular weight and sarcomere length both positively correlated with hip displacement severity, pathoanatomical changes identified in the myofibril may represent adaptations to help maintain a more uniform intrinsic passive muscle tension in response to spasticity and muscle shortening. For titin, this may manifest through the synthesis of a more compliant isoform despite the fact that mean molecular weights have not typically been shown to be different for children with CP [8, 24, 34]. Supporting this concept, in other research from our laboratory where passive stress generation in CP myofibrils was investigated, CP myofibrils were found to be more elastic than typically developing controls [35]. Despite this, at in vivo sarcomere lengths, myofibrils from CP muscles were found to be under substantially increased stress as compared to typically developing children. This alteration in compliance may be related to changes in the structural arrangement of titin filaments, but this was not investigated for the purposes of this study [12].

There were several limitations inherent to this pilot study. The first was the small sample size which made it difficult to make definitive statements regarding our findings. It was encouraging, however, that our findings were consistent with those concluded by others with larger sample sizes and that a post hoc power analysis proved favorable. Another limitation was the lack of a typically developing control group; however, our primary aim was to investigate the relationships between sarcomere characteristics and clinical dysfunction for children with CP rather than perform comparisons with typically developing children.

A final limitation is the analysis of gracilis alone as a proxy for the hip adductor group in CP. We agree that it would be beneficial to have analyzed not only gracilis but also adductor longus (and possibly adductor brevis) as two of the other most common muscles surgically treated for cases of hip displacement in CP [13, 36, 37]. As stated, the adductor longus biopsies harvested from the subjects in this study were used for the purposes of a separate analysis, the results of which have just been published. In this study from our laboratory, we found that there were no differences in sarcomere lengths, myofibril diameter, elastic modulus (i.e., stiffness), passive stress generation, and titin weight between adductor longus and gracilis [35]. As well, both adductor longus and gracilis (and sometimes adductor brevis) are the primary surgical targets for the treatment of hip displacement in CP [13, 37]. For these reasons, we feel that gracilis is a valid choice for analysis at the myofibrillar level to elucidate relationships to disease severity, even with the absence of adductor longus, given their similar pathophysiologic characteristics and clinical implication.

Acknowledgement should also be given that performing muscle biopsies at the end range of excursion rather than at a pre-determined joint angle may have induced some concerns as to the validity of our sarcomere length standardization during measurement. However, measuring sarcomere length at the end range for these muscles may reduce variability due to differences in inherent muscle slackness as discussed above.

## Conclusions

Understanding the relationship between hip displacement and pathophysiological changes at the sarcomere level is important to help in our understanding of muscle contracture development in CP. In this pilot study, we found that both titin size and sarcomere length were positively correlated to the severity of hip displacement. These important findings may represent adaptations intrinsic to the sarcomere that develop in response to the overall adductor muscle shortening associated with the development of hip displacement. To determine whether these changes are indeed compensatory mechanisms, rather than primary manifestations contributing to the development of hip displacement, requires further investigation. Understanding these relationships may provide the means to develop novel interventions that target these manifestations.

## Data Availability

The datasets used and/or analyzed during the current study are available from the corresponding author on reasonable request.

## Abbreviations

CP: Cerebral palsy
GMFCS: Gross Motor Function Classification System
MCPHCS: Melbourne Cerebral Palsy Hip Classification System
MP: Migration percentage
ROM: Range of motion
SEM: Standard error of the mean
SPSS: Statistical Package for the Social Sciences
